# Nature is (a) mine: conceptions of nature in the Dutch ecogenomics community

**DOI:** 10.1186/s40504-014-0010-y

**Published:** 2014-05-17

**Authors:** Sanne Van der Hout

**Affiliations:** Department of Philosophy and Science Studies, Radboud University Nijmegen – Faculty of Science, Institute for Science, Innovation and Society, P.O. Box 9010, 6500 GL Nijmegen, The Netherlands

**Keywords:** Ecological ethics, Ecological genomics, Metagenomics, Images of nature, Mining, Instrumental value, Valorisation

## Abstract

Every field of science, but especially biology, contains particular conceptions of nature. These conceptions are not merely epistemological or ontological, but also have normative dimensions; they provide an *ethos*, a framework for moral orientation. These normative dimensions, whilst often remaining ‘hidden’ and inarticulate, influence the way in which biologists practice their profession. In this paper, I explore what happens when different versions of these implicit normative frameworks collide. To do so, I will focus on a case study from the field of ecological genomics as it has evolved in one particular country, namely the Netherlands. During an important inaugural meeting, the director of one of the most sizeable Dutch ecogenomics centres gave a presentation in which he introduced the term ‘nature mining’. Part of the audience immediately embraced the term, but others were very reluctant. This mixed response is generally explained as a culmination of growing tension about the future direction of the field: due to new funding demands, a shift had occurred from fundamental research to research more interested in ‘valorisation’.

In addition to this current interpretation, I will argue that the turmoil caused by the use of the term ‘nature mining’ also reveals a more fundamental difference between the various parties involved in the Dutch ecogenomics community. This term is part of a vocabulary that emphasises the beneficial ‘goods’ produced by nature. Whereas part of the audience saw no harm in this commodification of nature, others had difficulties with the reduction of nature to a reservoir to be exploited using the latest technologies. I will conclude by arguing that, although at present, the core of Dutch ecogenomics research reflects a more or less instrumental attitude towards nature, the field also harbours other interpretations of nature as a significant and meaningful order. For instance, ecogenomics might further develop the image of land as a ‘collective organism’, as proposed by Aldo Leopold.

“Have you found the secret that I have lost?”

“Yes. You and the land are one.”

*Excalibur*

## Introduction

It is inconceivable to me that an ethical relation to land can exist without love, respect, and admiration for land, and a high regard for its value. By value, I of course mean something far broader than mere economic value; I mean value in the philosophical sense (Leopold 1949, 223).

In the 1940s, ecologist and forester Aldo Leopold (1887–1948) worked on a book that continues to stir millions of readers to this very day: “A Sand County Almanac”, published posthumously in 1949. The almanac contains a collection of essays in which the author sets forth his views on “the delights and dilemmas of one who cannot […] live without wild things” (Idem, vii). Leopold concludes his almanac with a plea for a ‘land ethic’, an admonition to enlarge “the boundaries of the community to include soils, waters, plants, and animals, or collectively: the land” (Idem, 204).

This study concentrates on one of the core messages of Leopold’s ‘land ethic’: every field of science, and especially biology, contains particular conceptions of nature. These conceptions are not merely epistemological or ontological; they have normative dimensions as well. They provide an *ethos*, a framework for moral orientation. These normative dimensions, whilst often remaining ‘hidden’ and inarticulate, influence the way in which biologists conduct their research and practice their profession. On certain occasions, however, normative aspects may suddenly rise to the surface, notably when moral clashes occur and biologists are confronted with conflicting images of nature (cf. Merchant 1989, 4). As environmental philosopher Martin Drenthen argues:

We are faced with a plethora of moral views of nature, all of which are deeply contingent. Our concepts and images of nature are the result of processes of interpretation, in which all sorts of cultural and historical influences play a part. […] It is only when our basic beliefs about nature are *challenged* by ‘moral strangers’ that we become aware of the particularity or perhaps even idiosyncrasy of our views (Drenthen 2005, 318).^a^

I will explore the normative dimensions of biology by means of a case study from the Dutch ecogenomics field. Ecogenomics – short for ‘ecological genomics’ – is an area of research which seeks to incorporate techniques and approaches originating from genomics in an ecological context. As ecological research and laboratory-based, molecular investigations traditionally occupied different areas within the biological sciences, this merging of ecology and genomics promises to “revolutionize our understanding of a broad range of biological phenomena” (Ungerer et al. 2008, 178).

During a memorable research meeting in February 2008, aimed at discussing the current state of Dutch ecogenomics research, a clash between ‘moral strangers’ took place. The participants in the meeting constituted a mixed audience: ecologists who took a more or less holistic stance to the study of ecological systems, molecular biologists with a preference “to work in controlled environments and with homogeneous well-defined genetic material” (Ouborg and Vriezen 2007, 13), industrial biotechnology experts looking for new market opportunities, and representatives of various intermediate positions. Bram Brouwer, director of one of the main Dutch ecogenomics centres, but also CEO of a private company operating in the fields of biotechnology and diagnostics, gave a presentation in which he introduced the term ‘nature mining’. Brouwer explained that the Earth’s ecosystems contain a huge number of valuable assets that are as yet unknown to us, such as antibiotics and enzymes. The emerging field of ecogenomics gives us the opportunity to ‘mine’ nature for these hidden goods (cf. Brouwer 2008).

The term ‘nature mining’ immediately threw the audience into disorder; part of the audience instantly embraced the term, whereas others had major reservations. The Dutch ecogenomics community has been a theatre of tensions for several years at this point. According to Roy Kloet and colleagues, they resulted from a disagreement about the future direction of the field: due to new funding schemes, a shift from fundamental research to research more interested in ‘valorisation’ – i.e. the process in which scientific knowledge is made profitable for society – had been initiated. Whereas the industrial partners welcomed the prospect of applications, some of the academic partners “fundamentally disagreed with a focus on economic valorization” (Kloet et al. 2013, 213–214).

In this paper, I will argue that we cannot fully grasp the turmoil caused by Brouwer’s presentation by reducing it to a strategic conflict about the field’s research focus; the tensions are also symptomatic of a more fundamental difference between the various parties involved. By introducing the term ‘nature mining’, Brouwer unintentionally pinpointed the fact that the members of the Dutch ecogenomics community endorse different, even conflicting conceptions of nature; this term is part of a vocabulary that emphasises the beneficial ‘goods’ produced by nature. Whereas part of the audience saw no harm in this “productivity outlook on nature” (Worster 1994, 271), others objected to the reduction of nature to a reservoir to be exploited using the latest technologies (Ouborg, interview, September 2012).^b^

In his work as a conservationist, Leopold noticed a ‘chasm’ similar to the one just described. In his view, the divide between different conceptions of nature was common to many specialized fields, such as forestry, agriculture, and wildlife management. In all these divides, Leopold argued, we can recognise the same basic ‘paradoxes’:^c^

man the conqueror *versus* man the biotic citizen; science the sharpener of his sword *versus* science the searchlight on his universe; land the slave and servant *versus* land the collective organism (Idem, 223).

I will use Leopold’s ‘paradoxes’ as a starting point to explore the different conceptions of nature within the Dutch ecogenomics community. I will start by giving an overview of the developments that preceded the aforementioned ecogenomics research meeting.^d^ Next, I will analyse why ‘nature mining’ turned out to be such an explosive and provocative term. Finally, I will argue that, although at present, the bulk of Dutch ecogenomics research reflects a more or less instrumental attitude towards nature, the field – in particular the metagenomic approach – also harbours other interpretations of nature as a significant and meaningful order, which could support a more humble and respectful approach to natural systems. A genomic approach to ecology might, for instance, cultivate the image of land as a collective organism, as proposed by Leopold.

## The establishment of the Ecogenomics Consortium

In 2002, the Dutch government established the Netherlands Genomics Initiative (NGI) as an independent taskforce to set up a “world-class genomics infrastructure”^e^ in the Netherlands. NGI called upon researchers to submit project proposals for the creation of a network of large-scale genomics centres. In response to this call, the Genomics for Ecology, Toxicology and Sustainable Technology Innovation Center (Gnettic) wrote a grant application letter envisioning the establishment of a centre of excellence in *ecological genomics*, “a novel, integrative field of science, combining ecology, microbiology, environmental & soil sciences and molecular biology” (Brouwer 2008, 1). The principal applicant of this programme was Bram Brouwer, director of BioDetection Systems, a company operating in the fields of biotechnology and diagnostics. Apart from Brouwer, the team consisted of various members of university research groups, for instance in the fields of animal ecology and molecular cell physiology.^f^

The participants submitted their letter of application, dated 23 September 2002, under the following heading: “Eco-genomics: the multidimensional analysis, experimentation and management of ecological systems for sustainable development” (Brouwer et al. 2002, 1). In this letter, the term eco-genomics (here still with a hyphen) was used for the first time in the Netherlands.^g^ The ambition of Gnettic was

to develop a set of genomics-based tools […] that can be used to analyze ecological systems, identify possible threats of contamination to the environment and human health, and to guide industrial production processes towards sustainable development” (Idem, 3).

The rationale for developing such a toolbox was that at the time, the level of understanding of ecological systems was inadequate for accurate predictions of responses to anthropogenic – i.e. manmade – disturbance. The biological instruments used in ecological assessments (biosensors, bioreporter systems, bioassays) were, in general, very labour-intensive. Moreover, they could only measure a limited number of targets at a given moment. The applicants argued that, in order to develop effective strategies for the sustainable production of animal and plant resources, major innovations were necessary. Genomics-based technologies enabled such innovations, “as they have the advantage that a multitude of targets can be evaluated at the same time with great responsiveness” (Idem, 3).

In analysing and managing ecological systems, Gnettic intended to apply two central approaches: *metagenomics* and the *organism-centred approach* (Marco 2010, preface).^h^ The first approach “enables us to study microorganisms in the complex communities where they actually live bypassing the need to isolate and culture individual community members” (Brouwer 2008, 1). In the 1990s, most microbiologists still assumed that the majority of microorganisms in a sample could be recovered by culturing them in the laboratory. An increasing amount of evidence nevertheless shows that “fewer than 0.1 % of the microorganisms in soil are readily cultured using current techniques. […] the other 99.9 % of soil microflora is emerging as a world of stunning, novel genetic diversity” (Handelsman et al. 1998, 245). By enabling the culture-independent genomic analysis of microbial populations, metagenomics “offers a window on an enormous and previously unknown world of microorganisms” (Handelsman 2007, 8).

The organism-centred approach seeks to improve our understanding of critical ecological interactions by focusing on the level of the individual organism. At the time of the Gnettic application, this approach was organised around classical laboratory-based model organisms, i.e. organisms with well-characterised gene expression patterns and large research networks around them, for instance the plant *Arabidopsis thaliana* and the nematode *Caenorhabditis elegans*, (Maher 2009, 695; Ankeny and Leonelli 2011, 316). By exposing the model to different environmental conditions (humidity, drought, etc.), the genes and gene functions that matter most in a given ecological interaction were identified (Ungerer et al. 2008). Because of the homology among organisms, the insights obtained from classical model organism studies were expected to provide insight into the biology of ecologically-interesting species as well: “We will exploit homologies across species to apply the insights obtained from models to other species, which are relevant for a wider range of environments than can be covered with the models only” (Brouwer et al. 2002, 5).^i^

The grant application of Gnettic was accepted by NGI and resulted in the establishment of the Ecogenomics Consortium (EC) in 2003. Brouwer was appointed as its director. The NGI-funded programme was entitled “Assessing the living soil: An ecogenomics approach to explore and unlock sustainable life-support functions of soils.” The consortium was to receive substantial funding, amounting to 1.8 million euros a year for the period of 2004–2009. Brouwer and his partners believed that the goals of EC would be best met by substantial investments in basic academic research: “research within the cluster is largely fundamental, for the simple reason that we know so very little about the living component of soil in particular” (NGI Annual Report 2002, 58). This focus on academic demands disappointed non-academic partners, “who felt they could contribute little to the composition of the board or to the EC’s research agenda. However, most did not complain as the EC funding was an additional opportunity to link their R&D activities to basic academic research” (Kloet et al. 2013, 212).

## From publication to product

In January 2008, NGI announced that its director Diederik Zijderveld was leaving. His departure implied a significant change for EC. Under the supervision of the academically oriented Zijderveld, NGI had focused on “creating a solid research infrastructure and a close-knit genomics community on the basis of excellent research” (NGI Annual Report 2008, 5). His successor Colja Laane, who had a background in industry, put a much stronger emphasis on ‘valorisation’, i.e. the process by which scientific knowledge is made profitable for society:

Our emphasis will be: from Publication to Product […]. All money and effort put into research must result in more applications. Valorisation is the motto, in terms of patents, licenses and new businesses.^j^

NGI’s shift in emphasis put the consortium’s members in a difficult position. The mid-term review of EC, which took place during the second half of 2006, had already pointed out that “achieving interdisciplinarity and […] realizing the societal mission” (Kloet et al. 2013, 213) were weaker points of the programme needing attention. The review committee had argued that, whereas the consortium’s achievements in terms of scientific excellence were quite impressive,^k^ it had difficulties employing “the knowledge to effect positive changes for society” (Veldhuis and Peels 2007, cited in Kloet et al. 2013, 214). In order to be considered for the second round of funding, EC had to implement NGI’s valorisation demands. This led to the establishment of the *Eco*genomics *In*novation *C*enter (ECO*L*INC), in which the ‘science-based’ focus of the 2004–2009 period was replaced by a more practical focus with a strong emphasis on “innovative aspects and valorization opportunities” (Brouwer 2008, 2). As Brouwer put it, “results and developments from the ongoing EC project have stimulated our ambition and increased our confidence that it is possible to assess and exploit nature’s vast hidden potential to develop sustainable applications in bio-based economy” (Idem, 1). ECO*L*INC received a follow-up grant of 3MEUR for 2009–2013 (compared to a budget of 11MEUR for 2004–2009).

The new focus of ECO*L*INC was clearly reflected in three of its main themes of investigation and valorisation. Firstly, the new programme sought to develop metagenomics and other ‘-omics’-based tools. The second theme dealt with the discovery of new functional capabilities of (un)cultivable microorganisms. Citing Brouwer again, “unleashing these hidden treasures will create a huge potential for applications in the fields of sustainable chemistry, alternative energy, in biorefineries, and in bio-construction materials” (Brouwer 2008, 2). Thirdly, ECO*L*INC focused on the development of “novel genomics-based cellular and whole organism test systems as alternatives for non-animal tests” (Idem, 2). Such alternative test systems were necessary in chemical industry for the safety assessment of large numbers of existing chemical compounds.

## The start of a new platform

The consortium’s move from basic ecogenomics research to a more practical approach was not welcomed unanimously. Whereas the industrial partners were happy with the “new market opportunities”, some of the academic partners “fundamentally disagreed with a focus on economic valorization” (Kloet et al. 2013, 213–214). To secure the “further development of basic and fundamental scientific knowledge” (Ouborg et al. 2009, 3), the latter started a parallel initiative, in cooperation with external research groups: the Platform Ecological and Evolutionary Genomics (PEEG), sometimes referred to as the National Program Ecological and Evolutionary Genomics (NP-EEG). PEEG was established in early 2007, a few months after a meeting in Soeterbeeck, aimed at getting a complete overview of ecogenomics research activities in the Netherlands. Initially, Brouwer and his allies were strongly opposed to the launching of PEEG, as they saw the new platform as a competitor. The members of PEEG, however, claimed that their programme should not be seen as a rival of ECO*L*INC, but rather as the continuation of the fundamental research project that was initiated by EC. PEEG’s financial sources were different from those for ECO*L*INC: for the 2009–2014 period, it received a funding of 1MEUR from the Netherlands Organisation for Scientific Research (NWO), enabling four PhD projects to be carried out (Van Straalen, interview, September 2013).

## Joining forces: the establishment of NERO

The establishment of PEEG did not put an end to the tensions within the Dutch ecogenomics community. To prevent the community from falling apart, the members of PEEG and ECO*L*INC decided to set up an umbrella organisation, by means of which the two programmes could be presented as ‘intertwined’, albeit with different orientations:

While both proposals have strong connections, they differ in the emphasis: NP-PEEG places emphasis on extending fundamental ecogenomics knowledge, as a requirement for developing applications, while ECOLINC places emphasis on the development of ecogenomics knowledge for biotechnology, while exploiting existing fundamental knowledge (Ouborg et al. 2009, 3).^l^

The umbrella organisation was called the Netherlands Ecogenomics Research Organisation (NERO). Its founding was not only considered a strategic move to calm things down, but also an effort to remain attractive for the financing parties. For indeed, to ensure continued funding, the Dutch ecogenomics community needed to come across as a robust and solid party (Ouborg, interview, August 2013). NERO described its mission as follows:

NERO will provide a platform function for ecogenomics, will act as co-ordinating organization, facilitating communication between the research field, financing agencies and end-users, will facilitate knowledge transfer in the form of workshops, thematic presentation days, and advanced international courses (Ouborg & Kammenga 2008, 27).

## Nature Mining

Even though NERO presented PEEG and ECO*L*INC as “two intertwined research programs” (Ouborg et al. 2009, 3), the friction between the two institutes became painfully clear during the very first National Ecogenomics Day (February 2008), the inaugural event in a series of annual meetings aimed at exploring the future of Dutch ecogenomics research. Moreover, it was on this occasion that NERO was to be officially introduced to the academic community at large. Position papers by leading experts from the Dutch ecogenomics community were presented, stressing the importance and the relevance of ecogenomics for various sub-disciplines of biology. Brouwer was one of the speakers. Faithful to the new strategy of NGI, he argued that Dutch ecogenomicists should put more emphasis on the ‘valorisation opportunities’ of their field of research. He suggested that one way in which ecogenomics research could be translated into viable opportunities, was by means of ‘nature mining’ (cf. Brouwer 2008). With this term, he referred to one of the two basic experimental approaches within the metagenomics field: the function-driven approach, in which microbial DNA is screened for potential applications in medicine, agriculture, and industry (Handelsman 2007).^m^ Natural ecosystems contain a huge number of valuable assets, such as antibiotics, vitamins, and enzymes. Function-based metagenomics enables us to ‘mine’ environmental samples – soil, sediment, groundwater – for these hidden goods (cf. Brouwer 2008).

Brouwer’s use of the term ‘nature-mining’ instantly revealed the existing discord within the Dutch ecogenomics community. Part of the audience – especially those with a background in industry – immediately embraced the term. They expressed their enthusiasm by persuading the organising committee to give Brouwer the opportunity to finish his talk (he had to cut short his speech due to a lack of time) at the end of the meeting. Others – notably the ecologists associated with PEEG – were very reluctant. In spite of their efforts to emphasise the importance of “extending fundamental ecogenomics knowledge” (Ouborg et al. 2009, 3), Brouwer now suggested ECO*L*INC’s strategy as a model for *all* Dutch ecogenomics research. Some of the attendants even had the impression that Brouwer wanted the term ‘nature mining’ as the new ‘brand name’ for research in the field of ecological genomics.

However, the tensions between the various research parties involved in NERO do not only give evidence of a strategic conflict concerning the (future) direction of Dutch ecogenomics research; they also show a more fundamental difference between NERO’s rank and file. NERO had united researchers coming from different branches of the biological sciences: ecologists with a “comprehensive way of looking at the earth’s fabric of life” (Worster 1994, x), molecular biologists with a more “mechanical picture of nature” (Idem, 40), industrial biotechnology experts interested in new research equipment for exploiting microbial systems, as well as representatives of various intermediate positions. All these parties brought along their own normative perspectives, their particular ways of interpreting the natural world as a morally significant order. This normativity usually remains hidden, but as a result of Brouwer’s presentation, and more specifically his use of the term ‘nature mining’, it suddenly came to the surface.

In the introduction, I explained that Leopold wrote about a ‘chasm’ between different images of nature as early as in the 1940s; he observed a divide which he considered to be common to many specialised fields, such as forestry, agriculture, and wildlife management. Each of these fields can be divided into a group that “regards the land as soil, and its function as commodity-production,” and a group that “regards the land as a biota, and its function as something broader” (Leopold 1949, 221). In all these divides, Leopold recognised the same basic ‘paradoxes’:

man the conqueror *versus* man the biotic citizen; science the sharpener of his sword *versus* science the searchlight on his universe; land the slave and servant *versus* land the collective organism (Idem, 223).

In the following sections, I will use Leopold’s ‘paradoxes’ as a guideline for exploring the different conceptions of nature existing within the Dutch ecogenomics community.

## Industrial mining

At the beginning of this paper, I explained that for some members of the Dutch ecogenomics community, the term ‘nature mining’ invoked an image of nature as a reservoir to be exploited using the latest technologies. As Joop Ouborg, co-founder of PEEG, put it: the term as such conveys a *technocratic* and *human-centred* image of nature. It echoes the question: how can we exploit nature to meet human needs? (Ouborg, interview, September 2012). In the field of environmental ethics, the interpretation of nature as a mere means to human ends is said to reveal an *instrumental* approach to nature (e.g. Rolston 1981; Curry 2006). Such an approach is based on the assumption that nature cannot have value independently of human needs and desires; it is thought to possess “meaning and value only when it is made to serve the human […] as a means to his or her ends” (Plumwood 2002, 109).

Why is the term ‘nature mining’ so strongly associated with an instrumental approach to nature? Obviously, this association largely revolves around the use of the term ‘mining’, i.e. the industrial process of extracting valuable minerals or other geological materials from the earth. Mining is one of the most pronounced examples of a process in which nature appears as a resource, as a *slave and servant* (cf. Leopold 1949, 223). By polluting “the ‘purest streams’ of the earth’s womb”, mining operations “have altered the earth from a bountiful mother to a passive receptor of human rape” (Merchant 1989, 38–39). In order to mine, trees and vegetation often have to be cleared. Moreover, large scale mining operations depend on industrial-sized machinery to extract the metals and minerals from the soil. Severely polluting chemicals, such as cyanide and mercury, are required to extract these valuable materials. Large amounts of waste materials are often discharged into rivers, streams, and oceans.^n^

The image of nature as a slave and servant became dominant during the Scientific Revolution and the rise of a market-oriented culture in early modern Europe. In her famous book “The Death of Nature” (1989), philosopher and historian of science Carolyn Merchant argues that in the Renaissance era, a different image of nature was still prevalent. Inspired by ancient Greek philosophers such as Anaxagoras (500–428 B.C.) and Theophrastus (370–278 B.C.), the Earth was viewed as a living organism and nurturing mother. This image had functioned as a normative constraint against the mining of Mother Earth: “One does not readily slay a mother, dig into her entrails for gold or mutilate her body” (Merchant 1989, 3). During the Scientific Revolution, this *vitalistic* image was replaced by a *mechanistic* view of nature: the Earth was no longer seen as a bountiful mother, but as an inanimate physical system. Merchant explains that the conception of the Earth as “a passive receptor” came to imply an approval of its exploitation, especially under the influence of Francis Bacon (1561–1626). She describes Bacon’s line of thought as follows:

Due to the Fall from the Garden of Eden […], the human race lost its ‘dominion over creation’. […] Only by ‘digging further and further into the mine of natural knowledge’ could mankind recover that lost dominion. In this way, ‘the narrow limits of man’s dominion over the universe’ could be stretched ‘to their promised bounds’ (Idem, 170).

Merchant thus claims that in Bacon’s view, God had not forbidden the ‘inquisition of nature’. Enslaving nature was, on the contrary, according to His plan: “Nature must be ‘bound into service’ and made a ‘slave’, put ‘in constraint’ and ‘molded’ by the mechanical arts. The ‘searchers and spies of nature’ are to discover her plots and secrets” (Idem, 169). Merchant explains that for Bacon, miners and smiths were the models for a new class of explorers, as

They had developed the two most important methods of wresting nature’s secrets from her, ‘the one searching into the bowels of nature, the other shaping nature as on an anvil’. […] For ‘the truth of nature lies hid in certain deep mines and caves,’ within the earth’s bosom (Idem, 171).

## Data mining

The term ‘nature mining’ cannot easily be disconnected from its association with disruptive mining practices. Yet, this association was amplified with other, similar elements in the vocabulary used by Brouwer. As mentioned before, he refers to the soil as a *treasure* at human disposal:

The application of metagenomics approaches […] will greatly extend our ability to discover hitherto hidden functional capabilities of (un)cultivable microorganisms. Unleashing these hidden treasures will create a huge potential for applications in the fields of sustainable chemistry, alternative energy, in biorefineries, and in bio-construction materials (Brouwer 2008, 2).

Another example of ‘tainted’ terminology was Brouwer’s description of ecogenomics as part of “the ‘Biotechnology for Nature’ field”^o^, as if it goes without saying that nature itself will benefit from our biotechnological interventions. Thus it was the “particular combination of terms, as well as the distinctive ways in which these terms [were] interpreted and related to each other” (Van Wensveen 1999, 11) that underlined the provocative and controversial view of nature in Brouwer’s speech.

Earlier, I explained that the term ‘nature mining’ was only rejected by part of Brouwer’s audience. NERO’s industrial partners, notably, received this term with warm enthusiasm. One possible explanation for this might be that they overlooked what this particular vocabulary meant for nature; the latter was merely seen “as the ‘environment’ or invisible background condition[…] against which the ‘foreground’ achievements of reason or culture […] take place” (Plumwood 1993, 4). Thus, in interpreting the term ‘nature mining’, the non-academic partners might have zoomed in on its positive impact on human progress*,* rather than on its destructive effects on nature. After all, the products of the mining industry have been, and still are, essential to human development.

Another explanation might be that the industrial partners – including Brouwer himself – had a different, more innocent and ‘neutral’ association in mind, namely ‘data mining’.^p^ Since the beginning of the digital information era, data overload has become a very common problem; we simply gather more data than we can process. The field “concerned with the development of methods and techniques for making sense of data” (Fayyad et al. 1996, 37) is known as ‘knowledge discovery in databases’ (KDD). Data mining officially refers to one of the steps in the knowledge discovery process, namely “the application of specific algorithms for extracting patterns from data” (Idem, 39). However, today the term is frequently used as a synonym for KDD, thus defined as “the nontrivial extraction of implicit, previously unknown, and potentially useful information from data” (Frawley et al. 1992, 58).

What is the image of nature that comes to mind when we interpret ‘nature mining’ as a derivative of ‘data mining’, i.e. as the extraction of previously unknown, and potentially useful information from large soil data sets? Contrary to industrial mining, data mining is a non-invasive approach: rather than extracting valuable ‘hardware’ (gold, coal, ore, petroleum, shale gas, etc.) from the Earth, it seeks to extract valuable ‘software’ (tangible knowledge) “adrift in the flood of data” (Frawley et al. 1992, 57). In an analogous manner, ‘nature mining’ attempts to screen large soil databases for useful information. Following this particular interpretation, the term ‘nature mining’ seems to be closely related to *biomimicry*, a scientific approach “that studies nature’s models and then imitates or takes inspiration from these designs and processes to solve human problems” (Benyus 2002, *preface*). However, although this interpretation does not evoke images of slavery or the ‘raping of mother earth’, the approach to nature still seems primarily instrumental. By comparing the soil to a database, “the natural world [is presented] as something that is passive and malleable in relation to human beings” (Rogers 1998, 244). The reduction of nature to a “passive object of knowledge” (Cheney 1992, 229) is one of the core themes in eco-feminist literature (e.g. Griffin 1995; Warren 2000; Plumwood 2002). Val Plumwood, an eminent Australian exponent of this particular movement, defines the interactions that originate from this reduction as *monological*, “because they are responsive to and pay attention to the needs of just one [namely the human] party to the relationship” (Plumwood 2002, 40). In a similar fashion, cultural theorist Richard Rogers argues that “objectification negates the possibility for dialogue […]. By transforming what exists into what is useful *to us* life is silenced” (Rogers 1998, 249–250 – author’s emphasis; cf. Evernden 1993, 88–94). Thus, even if we follow this more humble interpretation of Brouwer’s words, we still cannot escape the commodification of nature. Both analogies resonate the message: “Nature is (a) mine: it is ours!”

## After 2008

How has the Dutch ecogenomics field developed since the memorable research meeting in February 2008? As we have seen, NERO has had trouble keeping the different fractions of the Dutch ecogenomics community together from the very start. In June 2014, NERO will organise the 6^th^ National Ecogenomics Day with funding from NWO. Apart from this annual meeting, not much has been heard from NERO in recent years (Van Veen, e-mail correspondence, September 2013).

Compared to the situation in 2008, ECO*L*INC and PEEG have drifted even further apart. ECO*L*INC has put the valorisation issue even higher on its agenda. In 2010, it became part of the BE-Basic Foundation (Bio-based Ecologically Balanced Sustainable Industrial Chemistry), “an international public-private partnership that develops industrial bio-based solutions to build a sustainable society.”^q^ Brouwer is a key member of its leadership team, together with chemical engineer Van der Wielen (2012). Earlier, I pointed out that NGI had reserved a follow-up grant of 3MEUR for ECO*L*INC. As part of the BE-Basic programme, ECO*L*INC succeeded in obtaining an additional grant of no less than 18MEUR from the Economic Structure Enhancing Fund (FES), a policy-driven research fund. Officially, the BE-Basic programme would come to an end in 2015, but because of its success, it will continue until mid-2017.

The research conducted by BE-Basic is organised in ten ‘Flagships’, each addressing a significant scientific or socio-economic challenge. The seventh Flagship, entitled “High-throughput experimentation and (meta)genomic mining”, seeks to develop and apply “high-throughput approaches and tools to explore and mine the metagenome.”^r^ The vocabulary of the BE-Basic team reminds us of Brouwer’s speech during the first National Ecogenomics Day: nature appears as a resource for exploitation without constraint (cf. Plumwood 2002, 100). The term ‘nature mining’ has been replaced by ‘DNA-mining’, referring to the “search for enzyme variants in the total DNA pool found in […] soil and water samples.” Moreover, nature is presented in terms of *hunting*: “Nature will be the main hunting ground for [uncovering] novel enzymes with very special properties.”^s^

And what has become of PEEG, the fundamental research programme that was developed out of discontent with ECO*L*INC’s move towards valorisation? The five-year NWO-funding will expire in 2014. In 2010, members of PEEG started to collaborate with international colleagues in the European Collaborative Research programme ‘Ecological and Evolutionary Functional Genomics’ (EuroEEFG). The programme, which was terminated in May 2013, was funded by the European Science Foundation (ESF) and consisted of “eight collaborative research projects, spanning all kingdoms of life and various levels of biological organization (i.e. individuals, populations, communities, ecosystems).”^t^ The main goal of this collaboration was to bring about “a more successful scientifically-based management of ecological resources” (EuroEEFG programme 2011, 2).

Another ESF-funded project joined by (former) members of PEEG is the Conservation Genomics (ConGenOmics) Research Networking Programme. In this programme, the function of ecosystems is clearly understood “as something broader than mere economic value” (Leopold 1949, 223). This becomes apparent, for instance, in the statement that “conservation biology seeks to protect species and their habitats from the negative effects of [human-induced] changes” (ConGenOmics programme 2012, 2). Moreover, one of the aims of ConGenOmics is to “promote development of adequate conservation management programmes for endangered species at a European scale” (Idem, 7). ConGenOmics started in 2011 and will end in 2016.

## Hopes for the future

The ways in which the research programmes of ECO*L*INC and PEEG have developed up till now, remind us of one of the ‘paradoxes’ mentioned by Leopold. In the BE-Basic programme – currently the core of Dutch ecogenomics research – , science appears as the sharpener of the researcher’s sword (cf. Leopold 1949, 223), or, to stick to the vocabulary of the leadership team, as a hunter’s weapon. It is interesting to see that this specific vocabulary is embedded in a programme that seeks to contribute to the development of “new *sustainable* production processes” (Van der Wielen, presentation ESF Conference Towards a Sustainable Bio-Based Society, 6 December 2012 – my emphasis). Apparently, this instrumental language can be part of the rhetoric of sustainability.

The two ESF-funded programmes – especially ConGenOmics – are based on a different vocabulary. As they seek to improve our overall understanding of critical ecological interactions, science does not appear as a ‘weapon’, but rather as a *searchlight* for spotting complex ecological processes (cf. Leopold 1949, 223). Moreover, instead of understanding natural ecosystems as mere ‘commodity-production’ (Idem, 221), ConGenOmics explicitly seeks to protect natural ecosystems and its inhabitants from destructive human interventions.

In my view, there are various opportunities to include this more modest way of speaking in the BE-Basic programme, as well. Earlier, I explained that, in order to implement NGI’s valorisation demands, Brouwer and his research team increasingly concentrated on metagenomics. Compared to the organism-centred approach, this approach offers more opportunities for developing useful products and applications (e.g. medicines, vitamins, enzymes). At the present time, the usefulness of metagenomics to solve various complex human problems seems to encourage an instrumental approach to nature. However, this does not necessarily need to be so: the field also harbours other interpretations of nature as a significant and meaningful order, which could form the basis for a more humble and respectful approach to natural systems. For instance, metagenomics might cultivate the image of land as a *collective organism*, as has been proposed by Leopold; it shows us the interdependence of all life forms, or, to speak with Leopold, it shows us that we are all “member[s] of a biotic team” (Leopold 1949, 205). Traditionally, life is considered “to be organized around the pivotal unit of the individual organism” (O’Malley and Dupré 2010, 189). Metagenomics invites us to replace this ‘monogenomic’ conception by an organism- and species-free context: by demonstrating how genes “influence each other’s activities in serving collective functions”, the field encourages us to “explain and predict […] the behavior of the biosphere as though it were a single *superorganism* (Committee on Metagenomics 2007, 13 & 139 – my emphasis). Thus, for some practitioners, the field moves us “inexorably in the direction of a *Gaia*-like concept of the world” (Dupré 2007, 200, cf. Committee on Metagenomics 2007, 139 – my emphasis).^u^

**Figure 1:**
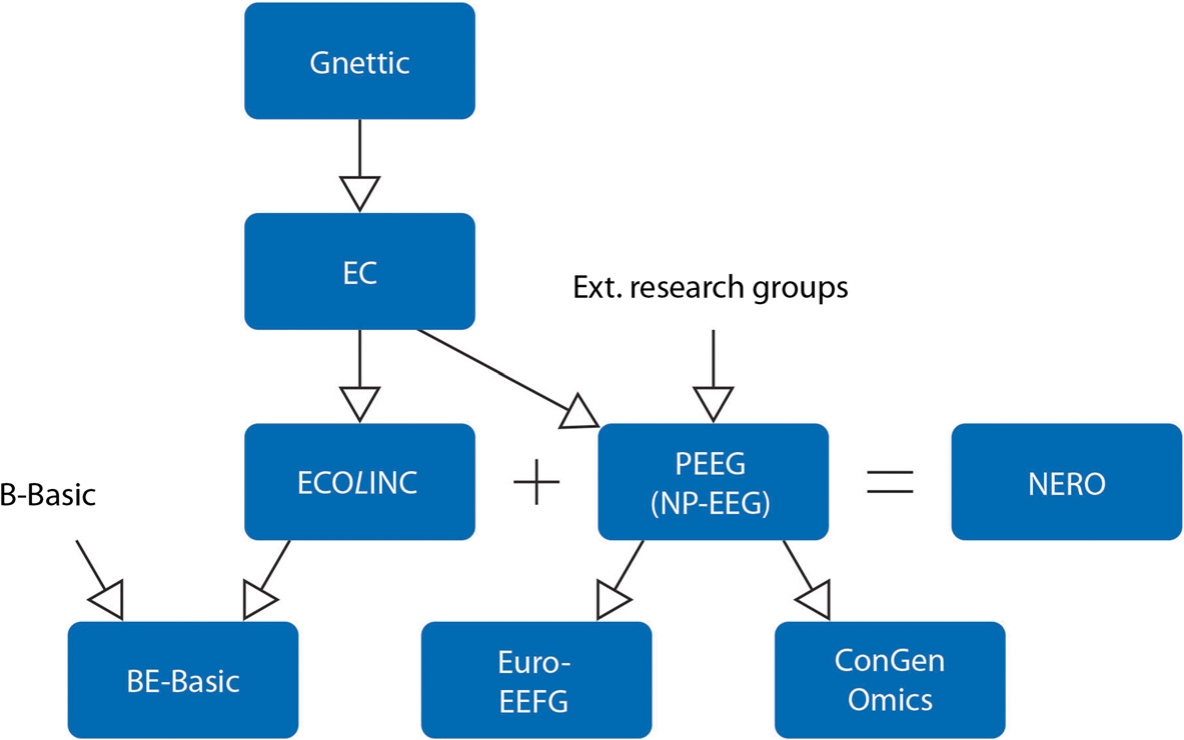
**Overview of the research parties involved in the Dutch ecogenomics community.**

Another way in which metagenomics might endorse a more respectful approach to natural systems is by confronting us with the crucial role of microbes in fulfilling all kinds of highly important human needs: the purification of drinking water, the development of new medicines, etc. (cf. Handelsman 2007, 8). From this angle, metagenomics could even encourage us to embrace an image of nature that is connected with the mythical image of the Earth as a *nurturing mother* (cf. Merchant 1989). We are thus reminded of the fact that we humans “are not only cultural beings but also natural beings, just as dependent on a healthy biosphere as other forms of life” (Plumwood 2002, 99). Therefore, one might say, even the field’s huge potential for products and applications does not necessarily need to go hand in hand with instrumental approaches to nature, but might, on the contrary, function as a basis for *respect*. But all this is no more than hope for the future. As Rogers argues: “The reconstruction of a different relationship to the environment in which we live requires radically alternative conceptions of humans, nature, material conditions, and discourse” (Rogers 1998, 268) (Figure 1).

## Endnotes

^a^In the work of Richard Rogers, we can find a similar argument: “Our theories do matter to the extent that they are […] produced in a particular historical context, existing in a web of ideological affiliations, and potentially effective in the social and natural worlds. We must therefore take them seriously – nor simply as more babel from the ivory tower, nor as ends in themselves, but as part of the ongoing construction of how the world, human beings, and social activity can and should operate” (Rogers 1998, 269).

^b^With the term ‘interview’, I refer to semi-structured philosophical conversations that I had with several of the key players in the Dutch ecogenomics field.

^c^Leopold’s use of the term ‘paradox’ appears to be somewhat misleading, as the views he describes seem to refer to ‘normal’ (i.e. non-paradoxical) oppositions.

^d^In concert with my interviewees, I have decided to mention the researchers and institutes involved, by name. This not only makes my analysis verifiable, but also enhances the tangibility and liveliness of the discussion.

^e^**Netherlands Genomics Initiative** [http://www.genomics.nl/Home/NGI/History.aspx] – accessed 13 September 2013.

^f^Other key participants were Nico van Straalen, professor of animal ecology at VU University Amsterdam; Hans Westerhoff, professor of microbial physiology, also at VU; Hans van Veen, head of the department of microbial ecology at the Netherlands Institute of Ecology; Jan Kammenga, assistant professor at the laboratory of nematology of Wageningen University.

^g^The term ‘ecological genomics’ was introduced by the Israeli biology professor Eviatar Nevo (1998). The abbreviation ‘ecogenomics’ first appeared in an official publication by marine biologist Robert Chapman (2001).

^h^For more on the metagenomic and organism-centred approaches, see Van der Hout (2013).

^i^As a result of technological advances (especially the introduction of next-generation sequencing methodology), the single-organism approach has recently succeeded in shifting its emphasis from research on traditional model species to ecologically-interesting species, e.g. the water flea *Daphnia Pulex*.

^j^**Netherlands Genomics Initiative** [http://www.genomics.nl/News%20archive/24%20April%202008.aspx] – accessed 13 September 2013.

^k^During the 2004–2009 period, EC generated 510 scientific publications (Kloet *et al*. 2013, 214).

^l^In his pre-proposal of ECO*L*INC, Brouwer also refers to the connection between the two ecogenomics programmes: “… a fundamental ecogenomics research program will be added to the ECO*L*INC proposal, funded by a separate source, namely the Earth & Life Science Program of the Netherlands Science Foundation” (Brouwer 2008, 2).

^m^The second approach, known as the *sequence*-*driven approach*, concentrates on the screening of microbial communities to reveal the overwhelming diversity of its members: “DNA from the environment of interest is sequenced and subjected to computational analysis. The metagenomic sequences are compared to sequences deposited in publicly available databases […]. The genes are then collected into groups of similar predicted function, and the distribution of various functions and types of proteins that conduct those functions can be assessed” (Handelsman 2007, 4).

^n^**Pink goes Green**

[http://www.dzpinkgoesgreen.org/blog.aspx?item=Blog/Blog%2052%20-%20Have%20You%20Heard%20About%20Responsible%20Jewelry.xml] – accessed 16 September 2013.

^o^**ECO*L*INC**

[http://www.ecogenomics.nl/index.php?option=com_content&task=view&id=11&Itemid=33&lang=english] – accessed 11 February 2014.

^p^Van Straalen explained that, because of the resistance evoked by the term ‘nature mining’, EC’s leadership team sometimes preferred to use the term ‘unlock’, e.g. in the title of the NGI-funded ecogenomics programme: “Assessing the living soil: An ecogenomics approach to explore and unlock sustainable life-support functions of soils” (interview, September 2013).

^q^**BE-Basic Foundation** [http://www.be-basic.org/] – accessed 2 September 2013.

^r^**BE-Basic Foundation** [http://www.be-basic.org/research/hte-metagenomic-mining.html] – accessed 2 September 2013.

^s^**BE-Basic Foundation** [http://www.be-basic.org/research/hte-metagenomic-mining/new-robust-enzymes-for-bioplastic-production.html – accessed 2 September 2013.

^t^**EuroEEFG** [http://www.nioo.knaw.nl/euroeefg] – accessed 16 September 2013.

^u^This Gaian perspective is not only applicable to natural ecosystems, but also to our human bodies. Metagenomics has demonstrated that our bodies consist for 90 per cent of microbial, rather than ‘human’ cells. As a result, metagenomics encourages as to conceive ourselves as collective organisms or ‘supraorganisms’ as well (Turnbaugh & Gordon 2008, 708; cf. O’Malley & Dupré 2007; Drenthen *et al*. 2009).

## Abbreviations

BDS: BioDetectionSystems
BE-Basic: Bio-based Ecologically Balanced Sustainable Industrial Chemistry
CSG: Centre for Society and the Life Sciences
EC: Ecogenomics Consortium
ECO*L*INC: *Eco*genomics *In*novation *C*enter
ESF: European Science Foundation
EuroEEFG: European Collaborative Research programme “Ecological and Evolutionary Functional Genomics”
FES: Economic Structure Enhancement Fund
NERO: Netherlands Ecogenomics Research Organisation
NGI: Netherlands Genomics Initiative
PEEG: National Program Ecological and Evolutionary Genomics
NWO: Netherlands Organisation for Scientific Research
PEEG: Platform Ecological and Evolutionary Genomics

## References

[CR1] Ankeny Rachel A, Leonelli S (2011). What’s so special about model organisms?. Studies in History and Philosophy of Science Part A.

[CR2] Benyus JM (2002). Biomimicry. Innovation Inspired by Nature.

[CR3] Brouwer B (2008). Sustainable Development of Bio-Based Applications in Chemical Industry. Grant proposal of the Ecogenomics Innovation Centre (ECO*L*INC).

[CR4] Brouwer B, Noomen GW, Louise EM Vet, Kropff Martin J (2002). Eco-genomics: the multidimensional analysis, experimentation and management of ecological systems for sustainable development. Letter of Intent of the Genomics for Ecology, Toxicology and Sustainable Technology Innovation Center (Gnettic).

[CR5] Chapman RW (2001). EcoGenomics – a consilience for comparative immunology?. Developmental and Comparative Immunology.

[CR6] Cheney J (1992). Intrinsic value in environmental ethics: beyond subjectivism and objectivism. The Monist.

[CR7] Committee on Metagenomics: Challenges and Functional Applications, National Research Council (2007). The New Science of Metagenomics: Revealing The Secrets of our Microbial Planet.

[CR8] Curry P (2006). Ecological Ethics: An Introduction.

[CR9] Drenthen M (2005). Wildness as a Critical Border Concept: Nietzsche and the Debate on Wilderness Restoration. Environmental Values.

[CR10] Drenthen M, Keulartz J, Proctor J (2009). New Visions of Nature. Complexity and Authenticity.

[CR11] Dupré J (2007). Processes of Life: Essays in the Philosophy of Biology.

[CR12] Evernden N (1993). The Natural Alien. Humankind and Environment.

[CR13] Fayyad U, Piatetsky-Shapiro G, Smyth P (1996). From Data Mining to Knowledge Discovery in Databases. AI Magazine.

[CR14] Frawley William J, Piatetsky-Shapiro G, Matheus Christopher J (1992). Knowledge Discovery in Databases: An Overview. AI Magazine.

[CR15] Griffin S (1995). The Eros of Everyday Life: Essays on Ecology, Gender and Society.

[CR16] Handelsman, Jo. 2007. Metagenomics and Microbial Communities. *Encyclopedia of Life Sciences*: doi:10.1002/9780470015902.a0020367.

[CR17] Handelsman J, Rondon Michelle R, Brady Sean F, Clardy J, Goodman Robert M (1998). Molecular biological access to the chemistry of unknown soil microbes: a new frontier to natural products. Chemistry & Biology.

[CR18] Kloet R, Hessels Laurens K, Marjolein BM Z, Jacqueline EW B, de Cock Buning T (2013). Understanding Constraints in the Dynamics of a Research Programme Intended as a Niche Innovation. Science and Public Policy.

[CR19] Leopold A (1949). A Sand County Almanac and Sketches here and there.

[CR20] Maher B (2009). Evolution: Biology’s next top model?. Nature.

[CR21] Marco D (2010). Metagenomics: Theory, Methods and Applications.

[CR22] Merchant C (1989). The Death of Nature: Women, Ecology and the Scientific Revolution.

[CR23] Nevo E (1998). Molecular evolution and ecological stress at global, regional and local scales: The Israeli perspective. Journal of Experimental Zoology.

[CR24] O’Malley Maureen A, Dupré J (2007). Size doesn’t matter: Towards a more inclusive philosophy of biology. Biology and Philosophy.

[CR25] O’Malley Maureen A, Dupré J, Marco D (2010). Philosophical Themes in Metagenomics. Metagenomics: Theory, Methods and Applications.

[CR26] Ouborg J, Kammenga J (2008). Ecogenomics in the Netherlands: exploration of opportunities and necessities. Vision document of the Netherlands Ecogenomics Research Organisation (NERO).

[CR27] Ouborg J, Vriezen W (2007). An ecologist’s guide to ecogenomics. Journal of Ecology.

[CR28] Ouborg J, Peter Van T, Rens V, van Veen H (2009). Research proposal National Program – Ecological and Evolutionary Genomics (NP-EEG).

[CR29] Plumwood V (1993). Feminism and the Mastery of Nature.

[CR30] Plumwood V (2002). Environmental Culture: the ecological crisis of reason.

[CR31] Rogers RA (1998). Overcoming the Objectification of Nature in Constitutive Theories: Toward a Transhuman, Materialist Theory of Communication. Western Journal of Communication.

[CR32] Rolston H (1981). Values in Nature. Environmental Ethics.

[CR33] Turnbaugh Peter J, Gordon Jeffrey I (2008). An Invitation to the Marriage of Metagenomics and Metabolomics. Cell Press.

[CR34] Ungerer MC, Johnson LC, Herman MA (2008). Ecological Genomics: Understanding gene and genome function in the natural environment. Heredity.

[CR35] Van der Hout S (2013). Bridging the lab-field divide? The ‘eco’ in ecological genomics. History and Philosophy of the Life Sciences.

[CR36] Van der Wielen L (2012). BBE Beyond Bioethanol. Presentation as part of the ESF Research Conference Towards a Sustainable Bio-Based Society, Amsterdam.

[CR37] Van Wensveen L (1999). Dirty Virtues: The Emergence of Ecological Virtue Ethics.

[CR38] Warren KJ (2000). Ecofeminist Philosophy: a Western Perspective on What it is and Why it Matters.

[CR39] Worster D (1994). Nature’s Economy: A History of Ecological Ideas.

